# Electrophysiological Characterization of Ts6 and Ts7, K^+^ Channel Toxins Isolated through an Improved *Tityus serrulatus* Venom Purification Procedure

**DOI:** 10.3390/toxins6030892

**Published:** 2014-02-28

**Authors:** Felipe A. Cerni, Manuela B. Pucca, Steve Peigneur, Caroline M. Cremonez, Karla C. F. Bordon, Jan Tytgat, Eliane C. Arantes

**Affiliations:** 1 Department of Physics and Chemistry, School of Pharmaceutical Sciences of Ribeirão Preto, University of São Paulo, Av. do Café, s/n, 14040-903, Ribeirão Preto, SP, Brazil; E-Mails: cerni@fcfrp.usp.br (F.A.C); manupucca@usp.br (M.B.P.); carolmc@fcfrp.usp.br (C.M.C.); karla@fcfrp.usp.br (K.C.F.B.); 2 Toxicology and Pharmacology, University of Leuven, O&N 2, Herestraat 49, P.O. Box 922, Leuven 3000, Belgium; E-Mails: steve.peigneur@pharm.kuleuven.be (S.P.); jan.tytgat@pharm.kuleuven.be (J.T.)

**Keywords:** *Tityus serrulatus*, neurotoxins, potassium channels, α-KTx, scorpion venom

## Abstract

In Brazil, *Tityus serrulatus* (Ts) is the species responsible for most of the scorpion related accidents. Among the Ts toxins, the neurotoxins with action on potassium channels (α-KTx) present high interest, due to their effect in the envenoming process and the ion channel specificity they display. The α-KTx toxins family is the most relevant because its toxins can be used as therapeutic tools for specific target cells. The improved isolation method provided toxins with high resolution, obtaining pure Ts6 and Ts7 in two chromatographic steps. The effects of Ts6 and Ts7 toxins were evaluated in 14 different types of potassium channels using the voltage-clamp technique with two-microelectrodes. Ts6 toxin shows high affinity for Kv1.2, Kv1.3 and *Shaker* IR, blocking these channels in low concentrations. Moreover, Ts6 blocks the Kv1.3 channel in picomolar concentrations with an IC_50_ of 0.55 nM and therefore could be of valuable assistance to further designing immunosuppressive therapeutics. Ts7 toxin blocks multiple subtypes channels, showing low selectivity among the channels analyzed. This work also stands out in its attempt to elucidate the residues important for interacting with each channel and, in the near future, to model a desired drug.

## 1. Introduction

*Tityus serrulatus* venom is composed of a mixture of components including antimicrobial peptides [1], hyaluronidase [2], peptides without disulfide bounds reporting vascular activity [3], such as hypotensins [1], and mainly neurotoxins with low molecular mass [4].These neurotoxins are the major toxins studied specially because of their interaction with Na^+^ or K^+^ channels and their relevance in the scorpion envenoming [5,6,7]. K^+^ channel toxins are particularly interesting since a number of different types of potassium channels exists and given the fact that a great number of potassium neurotoxins are selective, contributing to the discovery of new pharmacological effects and/or a novel drug.

Ts toxins are usually isolated by a combination of gel filtration, ion exchange chromatography and re-chromatography [8,9], resulting in three chromatographic steps before obtaining pure toxins. Ts venom has also been fractionated through reversed phase HPLC and re-chromatography on a C18 column to obtain pure toxin [10]. Another purification procedure used by our group is the fractionation of Ts venom using a CM-Cellulose-52 (CMC-52) column [11], resulting in time saved and the obtainment of pure Ts1, the major toxin of Ts venom that corresponds to approximately 16% of the eluted material. The other toxins from Ts venom are isolated by re-chromatography from the eluted fractions [2,12]. Another advantage of the CMC-52 chromatographic step is that the enzymes, such as hyaluronidase and proteases, especially metalloproteinases, are active at the employed chromatographic conditions. On the other hand, their activities are decreased or abolished during reversed-phase conditions [2,13]. In other words, Ts1 is the only toxin that can be isolated through only one chromatographic step, while the other toxins and enzymes need to be isolated through two or more chromatographic steps. 

Ts6 and Ts7—updated nomenclature by Cologna *et al.* [4]—are two of the potassium neurotoxins present in the Ts venom. Ts6 was first described as TsTX-IV by Arantes *et al.* in 1989 [11]. In contrast, its amino acid sequence was only revealed in 1999, characterizing Ts6 as a toxin with 41 amino acid residues and four disulfide bounds [14]. The same work also demonstrated that Ts6 blocks the high-conductance Ca^2+^-activated K^+^ channels in Leydig cells. Since Ts6 did not present identity with neurotoxins from the α-KTx families described so far, it was classified as the first toxin of the 12 subfamily, called α-KTx12.1 [15]. Butantoxin (BuTX), a neurotoxin with 40 amino acid residues and four disulfide bounds, was isolated from *Tityus serrulatus*, *Tityus bahiensis* and *Tityus stigmurus* through three chromatographic steps: gel filtration, cation exchange chromatography and re-chromatography on the same cation exchange resin [16]. BuTX is able to block reversibly *Shaker B* potassium channels and inhibits the lymphocyte T proliferation and IL-2 cytokine production [16]. Today, although both toxins Ts6 and BuTX are considered the same toxin [17], they are still classified as α-KTx12.1 (Ts6) and α-KTx12.2 (BuTX), since they derive from species from different geographic regions [18]. Recently, our group demonstrated that Ts6 stimulates the release of NO, IL-6 and TNF-α in J774.1 cells [19] and also presents a pro-inflammatory activity in mice [20].

Ts7, also known as TsTX-Kα, was first described as a toxin capable of blocking ^86^Rb efflux through the non-inactivating voltage-gated (delayed rectifier-type) potassium channel [21,22]. Classified as α-KTx4.1, Ts7 was considered a potential selective blocker in mammalian neurons culture [23] and it was able to block potassium current with high affinity in fibroblasts cells transformed to express Kv1.2 [24]. Further, using co-expression in pairs of three members of the *Shaker* subfamily of potassium channel α-subunits (Kv1.1, Kv1.2 and Kv1.4) expressed in *Xenopus laevis* oocytes, the heteromultimeric channel Kv1.1/1.2 appeared to be more sensitive to Ts7 than Kv1.2/1.4 [25]. In the past decade, Ts7 interaction with mouse Kv1.3 was studied using two expression systems (*Xenopus laevis* oocytes and naïve mammalian cells—L929). The same work demonstrated that Ts7 blocks the channel occluding the pore without changing its kinetic properties [26].

Therefore, although a lot of studies have been conducted with Ts6 and Ts7 toxins, a more complete understanding of their effect in blocking potassium channels is necessary to better understand their function in the envenoming syndrome or even discover a novel drug. In the present work, we describe an improved procedure of purification of the toxins Ts6 and Ts7 from *Tityus serrulatus* scorpion venom and investigate the interaction of both toxins in a wide screening on 14 subtypes of potassium channels, using electrophysiological experiments and voltage-clamp with two microelectrodes on *Xenopus laevis* oocytes.

## 2. Results

### 2.1. Purification and Biochemical Characterization of Ts6 and Ts7 Toxins

**Figure 1:**
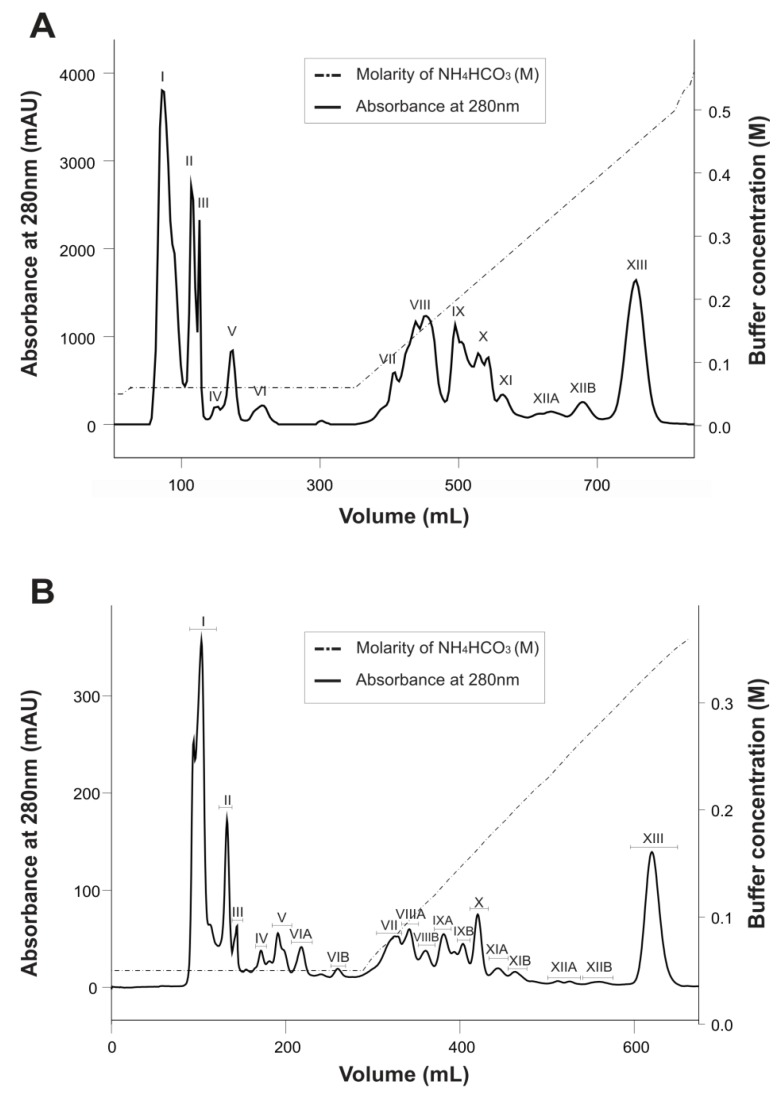
Elution profile of Ts venom on CM-cellulose-52 column. Absorbance was monitored at 280 nm. (**A**) Classical method of fractionation. *Tityus serrulatus* venom (200 mg) was dispersed in 2 mL of 0.05 M NH_4_HCO_3_ and the supernatant was fractionated on a column 2.5 cm × 63.0 cm, equilibrated with 0.05 M ammonium bicarbonate, pH 7.8 (Buffer A), at 4 °C. Flow: 0.3 mL/min. The dotted line represents the beginning of the convex concentration gradient of 0.01–1 M of ammonium bicarbonate (Buffer B). (**B**) Improved method of fractionation using a FPLC Äkta Purifier UPC-10 system. *Tityus serrulatus* venom (50 mg) was dispersed in 2 mL of 0.05 M NH_4_HCO_3_ and the supernatant was fractionated on a column 1.6 cm × 100.0 cm, equilibrated with 0.05 M ammonium bicarbonate, pH 7.8 (Buffer A), at 25 °C. Flow: 0.5 mL/min. The dotted line represents the beginning of the linear concentration gradient from 0% to 100% of 0.6 M of ammonium bicarbonate (Buffer B). Absorbance was monitored at 280 nm.

The Ts venom purification procedure, adapted to a FPLC system, exhibited an elution profile (Figure 1B) similar to the classical method (Figure 1A) on CM-cellulose column previously published [11]. The presence of Ts1, the major toxin, in the last peak on both methods (Fraction XIII) confirms this statement. However, the improved CM-cellulose method showed higher resolution of the peaks, which can be supported by the fractions VI, VIII, IX and XI that were divided into two subfractions—A and B (Figure 1B).

**Figure 2:**
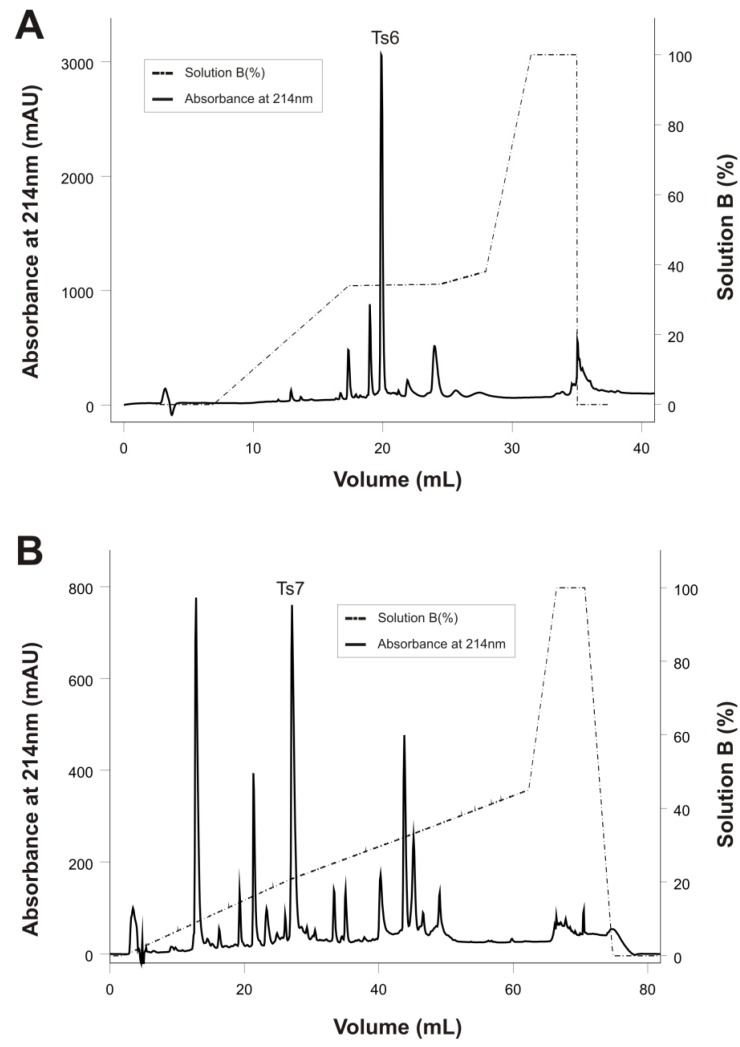
Reversed-phase FPLC of fractions X and XIIA resulting from the improved Ts venom fractionation procedure. The fractions were purified on a C18 column (4.6 mm × 250 mm, 5 µm particles) equilibrated with 0.1% (v/v) of trifluoroacetic acid (TFA). Adsorbed proteins were eluted using a concentration gradient from 0% to 100% of solution B (80% acetonitrile in 0.1% TFA), represented by the dotted line. Flow: 0.8 mL/min. Absorbance was monitored at 214 nm, at 25 °C. (**A**) Fraction X; (**B**) Fraction XIIA.

Ts6 was successfully obtained by reversed-phase chromatography from fraction X (Figure 2A) with some modifications of the method previously described [12]. Ts7 was obtained from the purification of fraction XIIA on a reversed-phase C18 column (Figure 2B). This toxin was previously purified using a reversed-phase chromatography of the fraction XI [26]. However, our group noted that Ts7 toxin was mainly found in fraction XIIA. 

The purity of Ts6 and Ts7 was confirmed by mass spectrometry and their primary sequence was verified by Edman degradation (data not shown).

The recovery percentage of Ts6 and Ts7 from the total venom is shown in Table 1.

**Table 1:** Fraction and toxin recovery

Fraction/Toxin	Column	Recovery %
X	CMC52	4.62
Ts6	C18	1.82
XIIA	CMC52	0.95
Ts7	C18	0.24

### 2.2. Electrophysiological Experiments

#### 2.2.1. Ts6 Toxin

**Figure 3:**
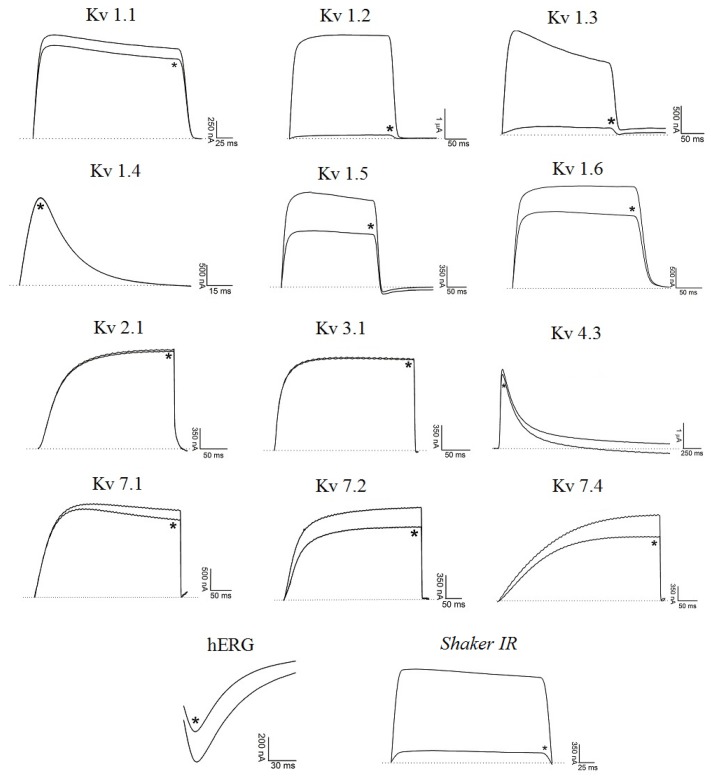
Blocking effect of Ts6 on 14 cloned potassium channels. Representative traces in the absence or presence (*) of 1 µM Ts6 are shown. Voltage protocol is detailed in Section 4.2 of Material and Methods.

**Figure 4:**
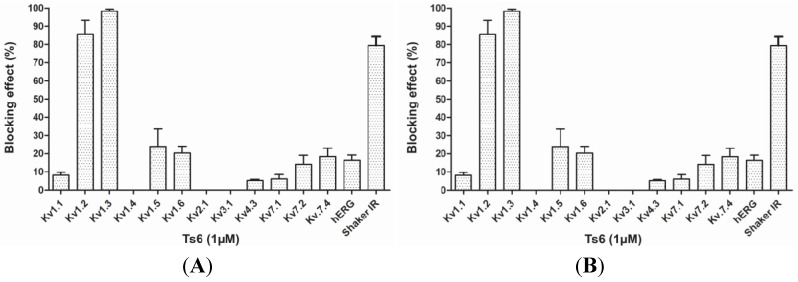
Blocking effect of toxins Ts6 and Ts7 on different types of potassium channels. (**A**) Ts6 blocking effect on 14 cloned potassium channels (*n* ≥ 3); (**B**) Ts7 blocking effect on 12 cloned potassium channels (*n* ≥ 3).

**Figure 5:**
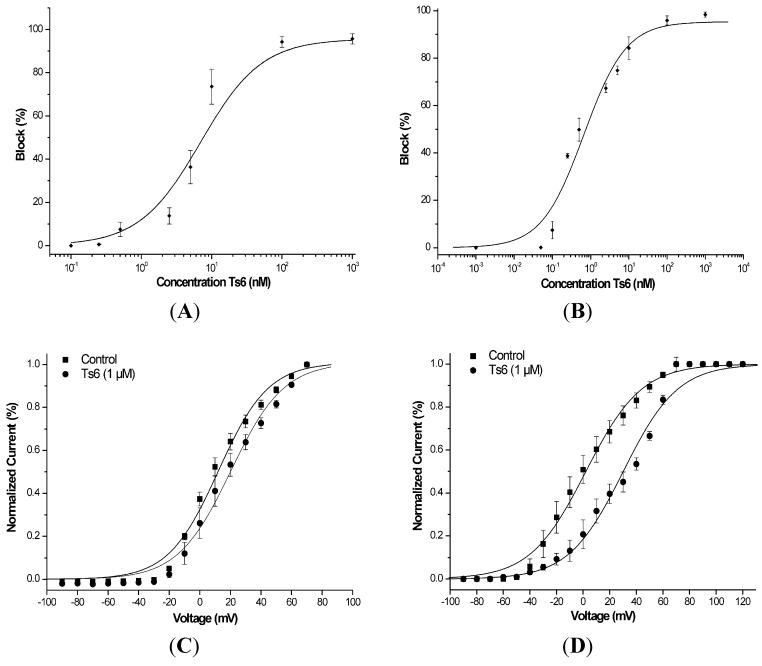
Functional features of Ts6 on Kv1.2 and Kv1.3 potassium channels. (**A**) Dose-response curve of Ts6 on Kv1.2 channels using Hill equation (for each tested concentration, *n* ≥ 3). (**B**) Dose-response curve of Ts6 on Kv1.3 channels using Hill equation (for each tested concentration, *n* ≥ 3). (**C**) Current/Voltage relationship on Kv1.2 in the absence (square) or in the presence of 1 µM Ts6 toxin (circle) (*n* ≥ 3). (**D**) Current/Voltage relationship on Kv1.3 in the absence (square) or in the presence of 1 µM Ts6 toxin (circle) (*n* ≥ 3).

Figure 3 demonstrates Ts6 electrophysiological traces on 14 different potassium channels. Ts6 showed a high and significant blocking effect on Kv1.2, Kv1.3 and *Shaker* IR with 86%, 98% and 79%, respectively. All the percentages of Ts6 blocking effect are represented in Figure 4A. Because Ts6 presents the higher blocking effect in Kv1.2 and Kv1.3, the dose-response curve was performed only in these cloned channels (Figure 5A,B). The IC_50_ values were 6.19 ± 0.35 nM (Hill = 0.9 ± 0.2) for Kv1.2 and 0.55 ± 0.20 nM (Hill = 1.8 ± 0.3) for Kv1.3. The current/voltage (I/V) curves (Figure 5C,D) showed that the inhibition on Kv1.2 channel in the presence of Ts6 is not associated with a change in the shape of the I/V relationship (V_1/2_ values yielded 12.2 ± 1.2 mV with a slope factor = 15.8 mV in control conditions and 20.3 ± 1.3 mV, slope factor = 16.8 after toxin application) (*p* < 0.05), whereas on Kv1.3 an inhibition of the current can also be in part due to the shift of the activation curve towards more positive potentials. The midpoint of activation for Kv1.3 channels shifted from 2.1 ± 1.1 mV, slope factor = 20.2 in control conditions towards 30.2 ± 1.6 mV and slope factor 19.7 after toxin application (*n* ≥ 3).

#### 2.2.2. Ts7 Toxin

**Figure 6:**
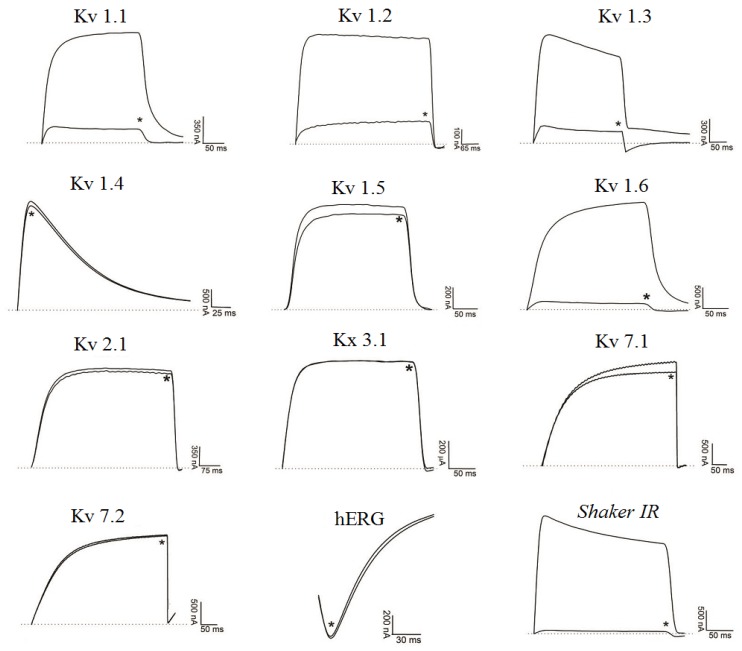
Blocking effect of Ts7 on 12 cloned potassium channels. Representative traces in the absence or presence (*) of 1 µM Ts7 are shown. Voltage protocol is detailed in Section 4.2 of Material and Methods.

Figure 6 presents Ts7 electrophysiological traces in 12 different potassium channels. A minor number of channels were tested due to the low recovery of Ts7 from the venom (see Table 1). The percentages of blocking effect of Ts7 are represented in Figure 4B. Ts7 showed a high and significant blocking effect on Kv1.1, Kv1.2, Kv1.3, Kv1.6 and *Shaker* with 85%, 91%, 89%, 94% and 97%, respectively. Since Ts7 presents high blocking effect in a variety of channels—considered not specific—the dose-response curves were not performed.

## 3. Discussion

Potassium channels (Kv) play an important role in calcium signaling, volume regulation, secretion, proliferation and migration in excitable and non-excitable cells [27]. Therefore, Kv channels toxins constitute potential drugs for the treatment of a variety of diseases like cancer, immunological, metabolic, neurological and cardiovascular disorders [28,29,30,31,32,33,34]. Additionally, studying isolated scorpion toxins can contribute to a better understanding of the scorpion envenoming syndrome and to the development of more effective antivenoms. 

The present work reports an improved method of purification and electrophysiological characterization of two α-KTx toxins—Ts6 and Ts7—from the venom of the Brazilian scorpion *Tityus serrulatus*. The improved method exhibits great reproducibility and a higher resolution than previously described, as well as appearing to be more practical [2]. The first step of venom fractionation on CM-cellulose column, at pH 7.8, is important in order to keep the enzymatic activities of metalloproteinases and hyaluronidase, which are decreased or abolished under reversed-phase chromatography conditions [2,13]. These enzymes are eluted on fractions different from those used to obtain Ts6 and Ts7. These α-KTx toxins were isolated by only two chromatography steps. 

Ts6 (previously named TsTX-IV, Butantoxin and α-KTx12.1) and Ts7 (known as TsTX-Kα and α-KTx4.1) were tested on a large set of Kv channels expressed in oocytes using two-microelectrode voltage clamp techniques.

**Figure 7:**
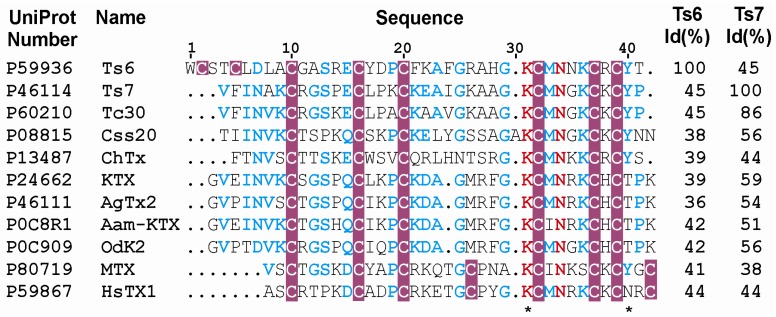
Multiple sequence alignment and Kv block effect of α-KTx toxins including Ts6 and Ts7. The aligment and percentage of identity Id (%) of 11 primary structures were created by ClustalW2. The figure was generated by ESPript and adapted in CorelDrawn13. UniProt accession numbers are followed by the toxins names. The highly conserved residues are in red. Cysteine residues are highlighted in pink. The amino acid residues in blue indicate low consensus and those not conserved are in black. The functional dyads are demarcated (*).

Ts6 showed a high blocking effect on Kv1.2, Kv1.3 and *Shaker* IR channels and was capable of blocking, with low efficiency, the channels Kv1.1, Kv1.5, Kv1.6, Kv4.3, Kv7.1, Kv7.2, Kv7.4 and h*ERG*. Concerning channel specificity, some key amino acids residues have been studied. In Ts6 primary sequence (Figure 7), the presence of Lys22 position can be responsible for the h*ERG* specificity, while the absence of Lys21 possibly justifies its low affinity, since Lys21-Lys22 is required for a high h*ERG* blockage [35].

Interestingly, Ts6 presented low effect on Kv1.1, but it was able to block with high efficiency the *Shaker* IR channel. *Shaker* was the first described gene encoding a potassium channel from *Drosophila* [36]. The result observed for Ts6 is an outstanding result, since Kv1.1 is homologous to the original *Drosophila Shaker* channel [37,38] and both channels present a hydrophobic/aromatic residue in the filter pore region [39]. Undoubtedly, Ts6 is not an impressive basic toxin (net charge = +3), being expected to have no effect on *Shaker* IR, Kv1.1, and Kv1.6 (Kv channels that display hydrophobic/aromatic residues in the pore filter), in line with the specificity of the TEA-binding (tetraethylammonium) on Kv channels [40]. Nonetheless, Ts6 induces *Shaker* IR high blockage, which led us to hypothesize that other amino acid residues may define its specificity. An individual feature of Ts6 toxin is that, unlike most of the Kv toxins from *T. serrulatus*, it presents an additional disulfide bridge, resulting in eight cross-linked cysteines. The importance of the number and arrangement of the cysteine residues can also provide a new framework to target Kv channels [41] and could be responsible for a new interactive mode of action of Ts6 toward *Shaker* IR channel. 

Ts6 showed high blockage on Kv1.2. This channel is expressed in the Central Nervous System (CNS) [27] and Kv1.2 knockout mice die from generalized seizures at post-natal day 17 [42]. Moreover, the work of Xie and co-authors shows that the Kv1.2 channels may be correlated with underlying human cerebellar ataxia disease [43]. Recently, it was demonstrated that Kv1.2 activation mediates the plasticity of dopamine release and offers a novel mechanism by which dopamine may be impaired in pathophysiological and neurological condition [44]. However, studies on this channel are considered still limited. Thus, Ts6 could be a useful tool for studying Kv1.2 channels.

Ts6 also blocked Kv1.3. This channel is expressed in T and B cells, macrophages, microglia, osteoclasts, platelets and CNS [27]. In human T and B lymphocytes, the voltage-gated potassium channel Kv1.3 and the calcium-activated potassium channel KCa3.1 are critically involved in the regulation of the membrane potential, calcium signaling and mitogen or antigen induced proliferation. It is known that expression of Kv1.3 changes dramatically as T and B cells differentiate from naïve into memory cells [27,45,46]. Additionally, autoreactive effector memory T lymphocytes, a cell subtype that overexpress Kv1.3, has been implicated in the pathogenesis of multiple sclerosis (MS), type-1 diabetes mellitus and psoriasis [27,47,48]. Since Ts6 presents a high blocking effect on Kv1.3, it could be a promising drug for the treatment of these autoimmune diseases.

Similarly to Ts6 (Figure 7), a toxin purified from the Brazilian scorpion *Tityus cambridgei* [49], Tc30 (45% of identity with Ts6), was able to block *Shaker* and Kv1.3 channel. HsTX1, a toxin from the scorpion *Heterometrus spinnifer*, also shares identity with Ts6 (44%) and was able to block Kv1.3 channels expressed in *Xenopus* oocytes with an IC_50_ of approximately 12 pM [50]. Our results shows that Ts6 can be considered an effective Kv1.3 blocker, acting in picomolar concentrations (IC_50_ of 0.55 nM or 550 pM), since OdK2, which is considered a potent immunosuppressive, blocks Kv1.3 with an IC_50_ of 7.2 nM [51]. Although Ts6 also blocks Kv1.2, many others Kv1.3 blockers that are attractive immunosuppressants were also not specific to the Kv1.3 channel; however, they appeared to block the channel at low concentration. The most potent known Kv1.3 inhibitor is the peptide ShK from the Caribbean sea anemone *Stichodactyla helianthus* [52]. ShK blocks Kv1.3 and suppresses proliferation of T_EM_ cells at picomolar concentrations (IC_50_ = 80 pM); however, ShK presents lower affinity for the neuronal Kv1.1 channel [53]. Margatoxin is also not specific for Kv1.3, since it also blocks at higher concentrations the Kvs 1.2, 1.1, 1.6 and 3.2. channels [54,55,56]. The same unspecificity was observed for the flavonoid Luteolin, a commercial nutriceutical [57]. Besides acting on Kv 1.3, Luteolin blocks the Kvs 1.2 and 1.5 channels [48]. Concerning Ts6, its IC_50_ shows to be 11.3-fold lower on Kv1.3 than on Kv1.2. Therefore, the specificity of Ts6 on Kv1.3, together with its picomolar affinity for the channel, makes it a promising drug for treatment of autoimmune diseases.

Ts6 exhibited 45% identity with Ts7 (Figure 7), which was capable of blocking, with low efficiency, the channels Kv1.5, Kv2.1, Kv3.1, Kv7.1 and h*ERG*. On the other hand, Ts7 presented a high block effect on Kv1.2, Kv1.3, *Shaker* IR, Kv1.1 and Kv1.6. Ts7 blockade of Kv1.3 had been previously described [26]. However, in that work, it was tested in a unique channel (mouse Kv1.3), masking the promiscuity of the Ts7 toxin. As described before, *Shaker* IR, Kv1.1 and Kv1.6 present a hydrophobic/aromatic residue in the filter pore region [39]. Ts7 is more basic (net charge = +6) than Ts6 (net charge = +3). In this case, Ts7 high blockage of *Shaker IR*, Kv1.1 and Kv1.6 is in agreement with the theory of TEA-binding [40]. Once Ts7 presents a high blocking effect on five mammal channels, it can be characterized as a nonspecific-toxin, preventing it from being used as a therapeutic drug. Besides its promiscuous nature, the amount of Ts7 in *T. serrulatus* venom is low (approximately 0.24% of the total protein against 1.82% for Ts6), which justifies why a dose-response assay was not performed and fewer channels were tested with Ts7 than with Ts6. Ts7 shows a high sequence identity with several toxins, which could explain why it acts on so many channels. Ts7 exhibits 62% identity with KTX-1, which is also classified as a promiscuous toxin since it blocks Kv1.1, Kv1.2 and Kv1.3 [58]. 

In spite of toxins recognizing different voltage-gated K^+^ channel subtypes, it has been evidenced that these peptides present in common some key molecular determinants [59]. An evolutionary tree indicates that several clusters of divergent peptides show preference for specific subtypes of channels. One example is the *functional dyad*, a key pair of amino acids in the toxin structure, which is described to be responsible for the interaction of toxins on potassium channels Kv1.x. This functional dyad is characterized by a lysine (K) and an aromatic/hydrophobic residue, usually tyrosine (Y), phenylalanine (F) or leucine (L), separated by 6–7 Å [60], see Figure 7. Both toxins studied—Ts6 and Ts7—present the *functional dyad* and, as expected, they act on potassium channels Kv1.x. Interestingly, the first empirical proof that the toxin projects its Lys27 into the pore, being responsible for disrupting potassium conduction in the selectivity filter, was published in 2013 [61], on the basis of co-crystal structure between Charybdotoxin (ChTX) and Kv channel. Further, it has already been reported that the sequences of Ts6 and Ts7 indicate that they could have a pharmacological target in Kv1.x channels [62,63]. 

Additionally, dimensional models of toxin-channel interactions provided evidence that other amino acid residues surrounding the functional dyad make important contacts with specific residues at the Kv1.x channels, which were defined as the *basic ring* residues [64]. These key determinants described fit well to the binding surface of various subtypes of potassium channels, except for *ERG*-channels, especially to the presence of an extra α-helix within the pore loop [65,66]. Some toxins with action on the *ERG-*channel were proposed to interact with the channel by an epitope formed by one hydrophilic and one hydrophobic patch, in separated regions of the toxins, which is called *two heads* interaction [67]. Besides the key determinants, a particular study realized with Ts6 indicated that the structural features of the toxin are pH-dependent. The authors showed that the presence of a His28 residue characterized by an unusually low pKa (5.2) allows Ts6 to maintain the structural and dynamics features that define its active state in a sufficiently wide pH range, which may be associated with toxin functionality [68]. Naranjo and Miller (1996) emphasized that M29 is a critical residue to the toxin interaction with the *Shaker* family channel [69]. Blanc *et al.* (1997) concluded that the interaction of the toxin with its receptor site depends on the whole charge distribution on the toxin [70]. 

Comparing the sequences (Figure 7), it is observed that ChTx has large, basic moieties (K11, R25 and K31) while Ts7 possesses smaller, uncharged residues (P11, A25 and G31) [71]. ChTx has a T8 which interacts with F425 on the *Shaker* channel [72], while Ts7 has a R8. The first disulfide bond which is observed only in Ts6 does not appear to confer stability to the protein [16]. However, the W1 ring and the possible hydrogen bonding of T41 ring oxygen to the H28 ring nitrogens are probably responsible for the reduced affinity of Ts6 for the *Shaker* B K^+^ channel [16].

In this work, Ts6 appears to be more specific for Kv1.3 channels, and can be considered a molecular model for production of immunosuppressive drugs, while Ts7 appears to block a range of channels being precluded to become a therapeutic drug. Recently, scorpion toxins specific for potassium channels revealed a great deal about channel structure and a number of human pathologies. The toxin bioengineering enabled the production of radio- and fluorescently-labeled toxins, chimera toxins and peptide cyclization, which are being used as biopharmaceuticals and therapeutic tools [73].

Although there are several potassium channel key determinants in the literature, previous studies analyzing common structural features present in 202 distinct scorpion sequences show that the information available on the various scorpion toxins is not sufficient to generate an encompassing theory capable of linking the structural with the physiological functions [74]. Therefore, additional structure-activity relationship studies on toxins are needed to understand the ion channel-toxin interaction. 

## 4. Experimental Section

### 4.1. Tityus Serrulatus Venom

Ts venom was obtained from the vivarium of the Faculdade de Medicina de Ribeirão Preto da Universidade de São Paulo (School of Medicine of Ribeirão Preto, University of São Paulo, Brazil), using electrical stimulation method [75]. About 100 scorpions were necessary to obtain 10 mg of venom with mucus (mean of three independent pool venoms). The mucus corresponds with 40%–50% of the crude venom.

### 4.2. Toxins Isolation

#### 4.2.1. Fast Protein Liquid Chromatography (FPLC) Using a CM-Cellulose-52 Column

*Tityus serrulatus* toxins Ts6 and Ts7 were purified using an improved method adapted from Pessini *et al.* [2]. Lyophilized Ts venom was moistened with 20 µL of ultrapure water and desiccated in vacuum for 6 hours. The desiccated step is important to improve the removal of mucus. Further, 50 mg of this was dispersed in 2 mL of 0.05 M NH_4_HCO_3_ buffer, pH 7.8, centrifuged at 10,015 *x g* for 10 min and the supernatant was stored at 4 °C. This procedure was repeated 2 times. The total supernatant (6 mL) was filtered through a 0.22-µm membrane (Millipore Ind. Com., Ltda, Brazil) and applied on a CM-celullose-52 column (1.6 cm × 100.0 cm) connected to a FPLC Äkta Purifier UPC-10 system (GE Healthcare, Uppsala, Sweden). At a flow rate of 0.5 mL/min at 25 °C, fractions were eluted with 140 mL of 0.05 M NH_4_HCO_3_, pH 7.8 (100% Buffer A) followed by a linear concentration gradient up to 0.6 M NH_4_HCO_3_ (100% Buffer B) during 440 mL of elution. This concentration (100% Buffer B) was held for 140 mL, declining abruptly (0 mL) to 0% of buffer B, reaching 100% of buffer A, which was maintained for 202 mL to reequilibrate the column. Absorbance at 280 nm was automatically registered.

The improved method was compared with the method previously published. As such, a protein fractionation was performed using 200 mg of *Tityus serrulatus* dried venom as described by Pessini and co-authors [2].

#### 4.2.2. Reversed-Phase (RP) FPLC Using a C18 Column

The fractions X and XIIA obtained from CM-cellulose-52 were used to obtain Ts6 and Ts7 toxins, respectively. RP-FPLC of each fraction was performed in an Äkta Purifier UPC-10 system (GE Healthcare, Uppsala, Sweden), using a 4.6 mm × 250.0 mm C18 column (Shimadzu Corp., Kyoto, Japan) equilibrated with 0.1% (v/v) trifluoroacetic acid (TFA) at a flow rate of 0.8 mL/min. The samples were eluted with steps of concentration gradient from 0% to 100% of solution B (80% acetonitrile in 0.1% TFA), at flow rate of 0.8 mL/min. Absorbance was monitored at 214 nm. Pure toxins were lyophilized and stored at −20 °C.

### 4.3. Biochemical Characterization of Toxins

The mass of Ts6/Ts7 toxins was measured by electrospray triple-quadrupole mass spectrometer (Quattro II, Micromass, Manchester, UK). The spectrum was processed using MaxEnt1 algorithm of MassLynx v3.3 software (Micromass, Manchester, UK).

The determination of amino acid sequence of Ts6 and Ts7 was performed by Edman degradation [76], on a Protein Sequencer model PPSQ-33A (Shimadzu Co., Kyoto, Japan).

### 4.4. Electrophysiological Experiments

#### 4.4.1. Potassium Channel Expression

cRNA for all Kv (rKv1.1, rKv1.2, rKv1.3, rKv1.4, rKv1.5, rKv1.6,rKv2.1, rKv3.1, rKv4.3, rKv7.1, rKv7.2, rKv7.4, h*ERG*, r*Shaker IR*) were synthesized from linearized plasmids using large-scale T7 or SP6 mMESSAGEmMACHINE transcription kit. The harvesting of oocytes from anesthetized female *Xenopus laevis* frogs was performed as previously described [77]. Oocytes were injected with 30–50 nL of the different channels using a microinjector (Drummond Scientific, Broomall, PA, USA). ND-96 solution was used for the oocytes incubation (in mM96 NaCl, 2 KCl, 2 MgCl_2_, 1.8 CaCl_2_, 5 HEPES, pH 7,4, supplemented with 50 mg/L gentamicin sulfate and 180 mg/L theophylline). The use of the frogs was in accordance with the license number LA1210239.

#### 4.4.2. Electrophysiological Measurements

Potassium currents were recorded using the two-microelectrode voltage-clamp technique at room temperature (18–22 °C).The recordings were processed by GeneClamp 500 amplifier (Axon Instruments, Foster City, USA) controlled by a pClamp data acquisition system (Axon Instruments, Foster City, CA, USA). Whole-cell currents from oocytes were recorded 1–5 days after injection. Currents and voltage electrodes had resistances from 0.5 to 1.0 MΩ and were filled with 3 M KCl. Currents were sampled at 2 kHz and filtered at 500 Hz using a four-pole low-pass Bessel filter. Leak subtraction was performed using a P/4 protocol. 

For the assays, 1 µM of each toxin (Ts6 and Ts7) was added directly to the recording chamber from a stock solution of ND-96 to obtain the desired final concentration. Immediately after adding the toxin to the chamber containing the oocyte, the bath solution was mixed to obtain a homogenous final concentration within a few seconds.

Currents from Kv1.1–Kv1.6 and *Shaker IR* were evoked by 500 ms depolarizations to 0 mV followed by a 500 ms pulse to −50 mV, from a holding potential of −90 mV. Currents traces from Kv2.1, Kv3.1 and Kv4.3 were evoked by 500 ms pulses to +20 mV from a holding potential of –90 mV. Currents from h*ERG* were elicited by applying a +40 mV prepulse for 2 s followed by a step to –120 mV for 2 s. Currents traces from Kv7.1, 7.2 and 7.4 were evoked by 3 s pulses to +40 mV from a holding potential of −90 mV. The voltage dependence of the relative current was fit by a Boltzmann function. The hill-coefficient was not held at constant values nor was the minimum and maximum of the fits constrained. Each experiment was performed at least 3 times (*n* ≥ 3). All data are represented as mean ± standard error. Data were analyzed using Clampfit version 8.1 (Molecular Devices, Sunnyvale, CA, USA), Microsoft Excel 2007 (Microsoft Corporation, Redmont, WA, USA), Origin Proversion 8.0 (OriginLab Corporation, Wellesley Hills, MA, USA). 

### 4.5. Toxins Sequence Alignment

The toxin sequences used and access number are deposited in the database Swiss-Prot. The amino acid sequences of the members of α-KTx subfamilies were retrieved from the Universal Protein Resource Knowledgebase [57]. The alignment and percentage of identity of the primary sequences of Ts6 and Ts7 were compared with other α-KTx toxins and created by ClustalW version 2 [78]. Only toxins presenting a high identity with Ts6 and Ts7 for which electrophysiological results have been reported in the literature were used in a sequence *versus* potassium-channel action comparison. The figure was generated by the ESPript server [79] and adapted in Corel Drawn version 13.

## 5. Conclusion

This work is a pioneer in the evaluation of Ts6 and Ts7 actions in 14 different channel types with the aim of determining which residues are important in the interaction with each channel. These studies could be essential for future applications of these toxins as drugs to treat channelopathies or as tools for structural and functional studies of potassium channels.

## Supplementary Data

### Toxins Sequence Alignment and Comparison of Kv Block Effect

The amino acid sequence of Ts6 and Ts7 was compared with other scorpion α-KTx toxin sequences deposited in the database (Figure 7). We chose scorpion toxins with identity greater than 35% with Ts6 and Ts7 for which electrophysiological studies have also been described in the literature (Table S1).

Ts6 presents the highest identity with Ts7 and Tc30 (both with 45%). Like Ts6, Ts7 is able to block Kv1.2 and Kv1.3. However, Ts7 also blocks other channels. Ts6 and Tc30 present blocking effect on *Shaker* IR channel and Kv1.3. The others toxins Css20 (38%), ChTx (39%), KTX (39%), AgTx2 (36%), Aam-KTx (42%), OdK2 (42%), MTX (41%) and HsTX1 (44%), although they present different electrophysiological effects from each other, all of them act on one or more of the channels for which Ts6 shows high effect (Kv1.2–1.3 or *Shaker* IR).

On the other hand, Ts7 presents a higher identity with the compared α-KTx toxin sequences than Ts6, also highlighting Tc30 (86%). KTX, Css20 and OdK2 also displays a high identity with Ts7. Compared to Ts6, the ten toxins analyzed present a higher identity to Ts7, which can probably explain why Ts7 acts on many channels. The other toxins ChTx (44%), AgTx2 (54%), Aam-KTx (51%), MTX (38%) and HsTX1 (44%), although presenting different electrophysiological effects from each other, all of them act on one or more of the channels for which Ts7 shows high effect (Kv1.1, Kv1.2, Kv1.3 or *Shaker* IR).

**Table S1:** Compared Kv block effect.

Name	α-KTx	Sensitive Kv channels/IC_50_	Insensitive Kv channels	Reference
Ts6	12.1	Kv1.2/6.19 nM, Kv1.3/0.55 nM, Shaker IR/*	Kv1.1, Kv1.4, Kv1.5, Kv1.6, Kv2.1, Kv3.1, Kv4.3, Kv7.1, Kv7.2, Kv7.4, hERG, KCa	#,[14]
Ts7	4.1	Kv1.1/*, Kv1.2/*, Kv1.3/*, Kv1.6/*, Shaker IR/*	Kv1.4, Kv1.5, Kv2.1, Kv3.1, Kv7.1, hEG	#,[24,25,26,71]
		native sqKv/20 nM,		
		cloned sqKv/10 nM		
		heteromultimer Kv1.2/1.1 SSM/10 nM		
		heteromultimer Kv1.2/1.4 EAM/0.7 nM		
Tc30	4.4	Kv1.3/* , Shaker B/*	-	[49]
Css20	2.13	Kv1.2/1.3 nM, Kv1.3/7.2 nM	Kv1.1, Kv1.4, Kv1.5, Kv2.1, Kv11.1 (hERG) KCa1, BKCa	[80]
ChTx	1.1	Shaker/*, Kv1.3/*, KCa	-	[81,82,83]
KTX	3.1	Kv1.1/1.1 nM–Kv1.2/25 nM–Kv1.3/0.1 nM	-	[58]
AgTx2	3.2	Shaker/*, Kv1.1/*, Kv1.3/*, Kv1.6/*	Kv2.1	[84]
Aam-KTX	3.12	Kv1.2/*, Kv1.3/*	Kv1.1	[58]
OdK2	3.11	Kv1.3/7.2 nM	Kv1.1, Kv1.2, Kv1.4, Kv1.5, Kv1.6, hERG , Shaker	[51]
MTX	6.2	Kv1.1/45 nM, Kv1.2/0.8 nM, Kv1.3/180 nM, Shaker/3.4 nM	-	[85,86]
HsTX1	6.3	Kv1.1/7 nM, Kv1.3/12 pM, IK/625 nM	Kv1.2	[50]

Notes: SSM: Single Subunit Model; EAM: Energy Additivity Model; #: Present Article: *: Data not reported.
